# Faster and improved 3-D head digitization in MEG using Kinect

**DOI:** 10.3389/fnins.2014.00326

**Published:** 2014-10-28

**Authors:** Santosh Vema Krishna Murthy, Matthew MacLellan, Steven Beyea, Timothy Bardouille

**Affiliations:** ^1^Biomedical Translational Imaging Centre (BIOTIC), IWK Health CentreHalifax, NS, Canada; ^2^Department of Diagnostic Radiology, Dalhousie UniversityHalifax, NS, Canada; ^3^Faculty of Computer Science, Dalhousie UniversityHalifax, NS, Canada

**Keywords:** magnetoencephalography (MEG), head position indicator (HPI), laser scanner, Microsoft Kinect, color recognition, alignment, localization

## Abstract

Accuracy in localizing the brain areas that generate neuromagnetic activity in magnetoencephalography (MEG) is dependent on properly co-registering MEG data to the participant's structural magnetic resonance image (MRI). Effective MEG-MRI co-registration is, in turn, dependent on how accurately we can digitize anatomical landmarks on the surface of the head. In this study, we compared the performance of three devices—Polhemus electromagnetic system, NextEngine laser scanner and Microsoft Kinect for Windows—for source localization accuracy and MEG-MRI co-registration. A calibrated phantom was used for verifying the source localization accuracy. The Kinect improved source localization accuracy over the Polhemus and the laser scanner by 2.23 mm (137%) and 0.81 mm (50%), respectively. MEG-MRI co-registration accuracy was verified on data from five healthy human participants, who received the digitization process using all three devices. The Kinect device captured approximately 2000 times more surface points than the Polhemus in one third of the time (1 min compared to 3 min) and thrice as many points as the NextEngine laser scanner. Following automated surface matching, the calculated mean MEG-MRI co-registration error for the Kinect was improved by 2.85 mm with respect to the Polhemus device, and equivalent to the laser scanner. Importantly, the Kinect device automatically aligns 20–30 images per second in real-time, reducing the limitations on participant head movement during digitization that are implicit in the NextEngine laser scan (~1 min). We conclude that the Kinect scanner is an effective device for head digitization in MEG, providing the necessary accuracy in source localization and MEG-MRI co-registration, while reducing digitization time.

## Introduction

Magnetoencephalography (MEG) is a non-invasive functional neuroimaging technology that maps brain activity by measuring magnetic fields generated from the electrical currents in the neurons of the brain (Cohen, 1968; Hämäläinen et al., 1993; Baillet et al., 2001). The electrical currents flowing in neurons are measured with multichannel, superconducting quantum interference devices (SQUIDs), which are installed in the MEG helmet (Hämäläinen et al., 1993). In modern whole-head sensor arrays, the localization accuracy combined with the high temporal resolution of MEG provides essential data for both fundamental brain research and clinical applications, such as pre-surgical mapping (Knake et al., 2006; Mäkelä et al., 2006; Stufflebeam, 2011). The accuracy of MEG for localizing brain activity is particularly valuable when MEG functional data are combined with anatomical data generated from magnetic resonance imaging (MRI). In this case, state of the art methods can localize the current source that is generating neuromagnetic activity with an accuracy of approximately 1 mm (Bardouille et al., 2012). However, the accuracy with which MEG functional and MRI anatomical data can be combined is dependent on how accurately the coordinate systems can be co-registered.

Accurate MEG-MRI co-registration is dependent on the appropriate definition of transformations between three coordinate systems. The fixed coordinate frame of the MEG device itself will be referred to as the “MEG scanner” coordinate system. As with MRI, the position of the MEG sensor array is fixed, and the participant's head is placed in the scanner. For this reason, the coordinate frame of the participant's head defines a second independent coordinate system, which will be referred to as the “MEG head” coordinate system. The MEG head coordinate system varies between individuals or even within an individual between scanning sessions (e.g., scans on subsequent days). Finally, the coordinate frame of the participant's head in the MRI scanner defines the “MRI head” coordinate system.

To align the MEG scanner and MEG head coordinate systems, head position indicator (HPI) coils are attached to the participant's head prior to scanning. The 3-D locations of the HPI coils are digitized in the participant's head coordinate system before the scan, generally using the Polhemus electromagnetic digitization system (Polhemus Incorporated, VT, USA). As well, anatomical landmarks, such as the nasion and pre-auricular points, are digitized to facilitate MEG-MRI co-registration. During the scan, the HPI coils emit known magnetic fields. The location of these HPI coils in the MEG device coordinate system can be determined by fitting a magnetic dipole to the measured magnetic fields (Incardona et al., 1992; Fuchs et al., 1995). A transformation between MEG scanner and MEG head coordinate systems can then be obtained based on the estimated locations of the HPI coils in each coordinate system. Similarly, the anatomical landmarks digitized prior to the MEG scan can also be manually identified on the MRI. A transformation between MEG head and MRI head coordinate systems can then be obtained based on the estimated locations of the anatomical landmarks in each coordinate system. Acquiring more digitization data on the shape of the head and face helps better constrain this second transformation.

The transformations between the three essential coordinate systems are strongly dependent on the accurate localization of the HPI coils and anatomical landmarks. Previous work has compared the performance of two digitization systems for accurately defining these transforms (Bardouille et al., 2012). Performance was compared between the commonly used Polhemus device and a high-resolution 3-D laser scanning digitization system (NextEngine, CA, USA). It was shown that the accuracy for localizing HPI coils with the Polhemus system was 1.9 mm, which limits the accurate alignment of MEG scanner and head coordinate systems. This error in HPI localization leads to a current source localization accuracy of 2.42 mm. This source localization accuracy was 1.38 mm poorer than was achieved using the laser scanner, which localizes the HPI coils to within 0.8 mm. Similarly, inaccurate localization of the few anatomical landmarks used for aligning the MEG and MRI head coordinate systems negatively impacts on the MEG-MRI co-registration. While acquiring additional points can ameliorate this negative impact, the Polhemus device can only acquire approximately 1 location per second, which limits data collection in a reasonable amount of time. In comparison, automated alignment of the high-resolution laser images of the face with the anatomical MRI reduces MEG-MRI co-registration error by more than a factor of three (Bardouille et al., 2012; Hironaga et al., 2014), as compared to Polhemus digitization combined with manual identification of the anatomical landmarks on the MRI.

In the ideal case of a stationary participant, high-resolution 3-D head digitization using the laser scanner leads to a significant improvement in localization accuracy over the Polhemus system. However, digitizing with the NextEngine laser scanner requires that the participant remain still for approximately 1 min while each laser scan takes place. While alternate laser scanning technologies are available with shorter scan times, these devices may be too expensive for use in many laboratory environments (Hironaga et al., 2014). Without reduced scan times, laser scans will be blurry and warped for participants who have difficulty remaining still for this period of time. As such, an alternate technology for high-resolution optical 3-D head digitization is required.

The Kinect sensor (Microsoft Corp., Redmond, WA, USA) has the potential to overcome the difficulties and challenges faced by the Polhemus and NextEngine devices. The Kinect is a motion-sensing device that uses infrared (IR) technology to capture the 3-D environment. The Kinect sensor has been used in a wide range of applications and has depth accuracy in the order of millimeters (Wilson, 2010; Chang et al., 2011; Khoshelham and Elberink, 2012). The sensor can capture both color and depth data simultaneously at a frame rate of up to 30 fps, with each frame co-registered to the previous frame in real-time using the Kinect Fusion Explorer software, packaged in the Microsoft Kinect for Windows Software Development Kit (SDK) and Developer Toolkit v1.8 (http://www.microsoft.com/en-ca/download/details.aspx?id=40278). This online co-registration means that an increased point density and surface area can potentially be obtained by moving the Kinect around the participant's head. Further, the Kinect can potentially capture the movement of the participant's head during the digitization process.

The aim of the current study was to compare the performance of the Kinect sensor with the Polhemus and laser scanner digitization systems for source localization accuracy and MEG-MRI co-registration. Source localization accuracy was determined using a calibrated phantom, and MEG-MRI co-registration error was determined based on MEG and MRI scans with human participants. We hypothesized that the source localization accuracy and MEG-MRI co-registration accuracy with the Kinect sensor would be equivalent to the accuracy of the laser scanner, and better than the Polhemus.

## Materials and methods

### Phantom study—source localization accuracy

**Figure 1:**
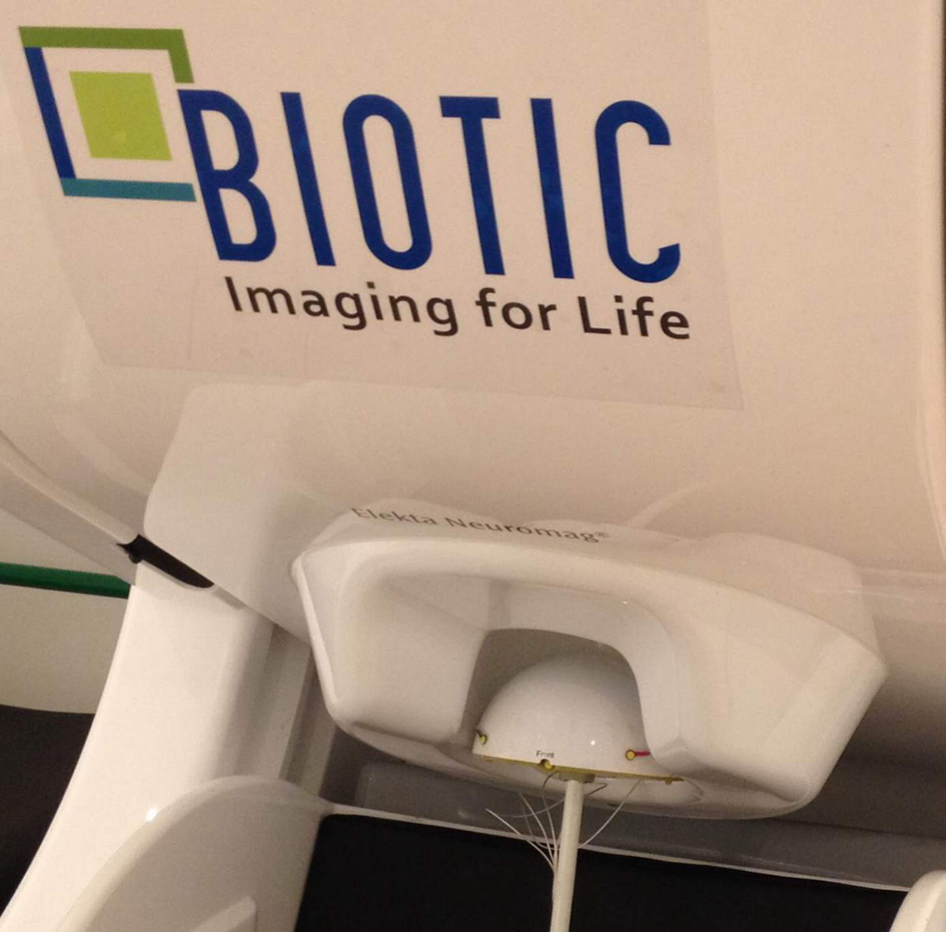
**Calibrated phantom inside the MEG scanner**.

To determine the source localization accuracy, we used a calibrated phantom (Elekta Neuromag, Finland), which contains 32 current sources at known locations (Figure 1). Four HPI coils were attached to the surface of the phantom to simulate common placements on the human head (on the forehead and near each ear). The digitization process with each of the three devices was performed by the same operator.

#### Phantom digitization

Polhemus 3-D digitization was performed by mounting the phantom on a nonmagnetic stand outside the magnetically shielded room (MSR). The locations of landmark points and the HPI coils were acquired by button presses on the Polhemus digitizing stylus. An additional reference sensor was attached to the phantom to correct for any movement of the phantom during the digitization process.

Laser 3-D digitization was performed by installing the phantom on a tripod mount. Yellow stickers were attached on the HPI coils before the digitization to aid in automatically identifying the coils offline. Distinct markers were also attached on the surface of the phantom to aid in image alignment, as the phantom was axially symmetric. Following this, eight individual scans were taken of the phantom at equal angles around the phantom's circumference. Individual scans were aligned manually using the vendor supplied laser scanner software, clusters of yellow points were identified, and the 3-D location for each cluster was automatically determined to localize each HPI coil.

Kinect 3-D digitization was also performed with the phantom in the tripod mount using the Fusion software. The Fusion software was modified using Microsoft Visual Studio Express 2013 to complete a real-time image erosion technique that removed artifacts near the boundary of objects in the depth image. The Fusion software reads both depth and color images from the Kinect device and maps the entire depth frame onto the color space to obtain colored 3-D points. Because the color image and depth image data come from separate sensors on the Kinect device, the background colors in the image near the edge of a foreground object (i.e., edges in the depth image) can be mapped onto the edge of the foreground object. As a result, some color smearing occurs in the final 3-D image, wherein objects close to the camera pick up color from the background.

Using the default Fusion software, visual inspection of captured models showed that the colored stickers appeared distorted. This distortion was eliminated using the erosion function. For each real-time depth image, each pixel that was part of the background (outside of the range of 400–1000 mm from the camera plane) was set to zero. An erosion function with a square 15 × 15 pixel kernel was then applied to the image such that pixels at the boundary of a close object were set to zero. Specifically, a copy of the depth image (i.e., new depth image) was generated. The erosion kernel was then centered over each pixel in the original depth image. For each pixel, if the centered pixel in the original depth image had a value of zero, then all pixels in the new depth image covered by the kernel were set to zero. The function had no effect if the center pixel in the original depth image had a non-zero value. To optimize the erosion function for processing speed, the function also had no effect if the center pixel had value zero in the original depth image but the kernel had already overlaid this pixel. The new (eroded) depth image was then used in further analysis, and applied as a mask to the color images to eliminate edge effects on close objects.

#### Source localization accuracy

In order to measure the phantom source localization accuracy, we installed the phantom under the MEG sensor array (Elekta Neuromag, Finland) following digitization using a non-magnetic mount. HPI coil locations were measured continuously during the MEG scan by activating each coil with a sinusoid of known frequency and magnitude. At the same time, a current source was activated for two cycles of a 20 Hz sinusoid at a magnitude of 500 nAm. MEG data was collected at 1000 Hz with a low-pass filter at 330 Hz. The source activation was repeated 100 times for each of the current sources. Five identical scans were performed.

For each scan, the MEG data for each source activation was isolated as a single trial. All 100 trials for a given current source were averaged together to generate an inter-trial average in which magnetic fields with no temporal correlation to the activity of interest were attenuated. To further improve the signal-to-noise ratio, environmental noise was removed from the MEG data using signal-space separation (Taulu et al., 2004), and the data was low-pass filtered at 40 Hz. For each current source, the magnetic field measurement at each MEG sensor at the peak of the first cycle of the sinusoid defined the known magnetic field. The current source was localized by modeling the source as an equivalent current dipole (Hämäläinen et al., 1993). The location, orientation and magnitude of the modeled dipole was permuted using a recursive algorithm to reduce the least-squares error between the known and modeled magnetic fields. The current source locations were estimated using HPI coil locations determined by each of the three devices. The known locations of the current sources were subtracted from the estimated source locations for each current source, scan and device to give an estimate of the displacement from the known location. The mean and standard error of the displacement from the known locations was determined across all the repeated scans to estimate the source localization accuracy for each of the device at each current source location. Finally, a repeated measures analysis of variance (ANOVA) with three factors (digitization device, current source location, and dimension) was performed to determine whether there was a significance difference between the devices for source localization accuracy (*p* < 0.01).

### Human study—MEG-MRI co-registration accuracy

#### Data acquisition

Five healthy participants were enrolled in the MEG study, which had full research ethics approval from the Research Ethics Board at the IWK Health Centre and Capital District Health Authority. All participants were informed about the study, the publication of their images and results and signed a consent form before participating. Each participant underwent head digitization by the same operator with each of the three digitization systems, and a high resolution T1-weighted MRI scan with a voxel size of 1 mm^3^.

Before digitization, four HPI coils were attached on the participant's head—two on the forehead and one in front of each of the ears. Colored markers were attached on the HPI coils (yellow) and the anatomical landmarks (nasion and pre-auricular points; blue) to aid in the identification process for the laser and Kinect devices.

To perform the Polhemus digitization process, the subject was seated in a wooden chair with their head within 50 cm of the electromagnetic field transmitter. A reference sensor was attached using a headband to correct for any movement during the digitization process. The location of the HPI coils, anatomical landmarks, and also approximately 150 additional points across the entire head were digitized using the Polhemus stylus sensor. The additional points were collected in the continuous collection mode, which acquires approximately 1 point per second. Digitization data was collected from the Polhemus in approximately 3 min per person.

The laser scanner digitization was performed once the Polhemus digitization process was completed. Three laser scans, one on the front and one on each side of the face, were digitized with each laser scan acquisition being completed in 1 min. Digitization data was collected from the laser scanner in approximately 3–4 min per person. Individual scans were aligned manually.

The Kinect digitization was achieved with one continuous Kinect scan. The operator performed the scan by holding the Kinect at a distance of approximately 50 cm from the participant's head, and walking around the participant. The arc of the scan was approximately 180 degrees, from ear to ear. As above, the co-registration of depth and color images acquired at 30 frames per second occurred in real-time to build a 3-D model of the head, including the erosion algorithm to correct for image artifacts occurring at object edges. Digitization data was collected from the Kinect scanner in approximately 1 min per person.

#### MEG-MRI co-registration

MEG-MRI co-registration accuracy was compared between digitization systems using a previously published approach (Bardouille et al., 2012). First, the scalp surface was extracted from the MRI data using a surface segmentation algorithm with 5% threshold intensity on the maximum voxel intensity (implemented in the SPM2 analysis package) (Ashburner and Friston, 1997). A “priming” (Euclidean) transformation (constant for each device) was then applied to the digitization data from each of the three devices to place the MRI facial surface data and the digitization data from each device in a similar coordinate frame. Following this, for each participant and each digitization system, a K-D tree (Bulan and Ozturk, 2001) iterative closest point (ICP) algorithm (Besl and Mckay, 1992) with point-to-plane error minimization (Chen and Medioni, 1992) was used for automatic co-registration of MRI and digitization data. For the laser scanner and Kinect sensor, the scalp surface and digitization data were restricted to include the face data above the upper lip and below the hairline only. Previous work has shown that digitization data with high spatial resolution from the face alone is sufficient to achieve optimal co-registration accuracy over the whole head (Koessler et al., 2011; Bardouille et al., 2012). In all cases, the ICP algorithm converged within a maximum of 25 iterations. MEG-MRI co-registration was visually verified using the vendor supplied MRI viewing software (MRI Lab, Elekta Neuromag, Finland). For each co-registered point in each digitization dataset, the co-registration accuracy was calculated as the distance between the digitized point and the nearest point on the MRI-derived scalp surface. The mean co-registration accuracy and standard error was also calculated across the digitization dataset for each subject and each digitization system. The inter-subject mean and standard error in mean co-registration accuracy was calculated to provide a comparison between digitization systems across participants. Finally, a repeated measures ANOVA with one factor (digitization device) was performed to determine whether there was a significance difference between the devices for MEG-MRI co-registration accuracy (*p* < 0.01).

## Results

### Phantom study

**Figure 2:**
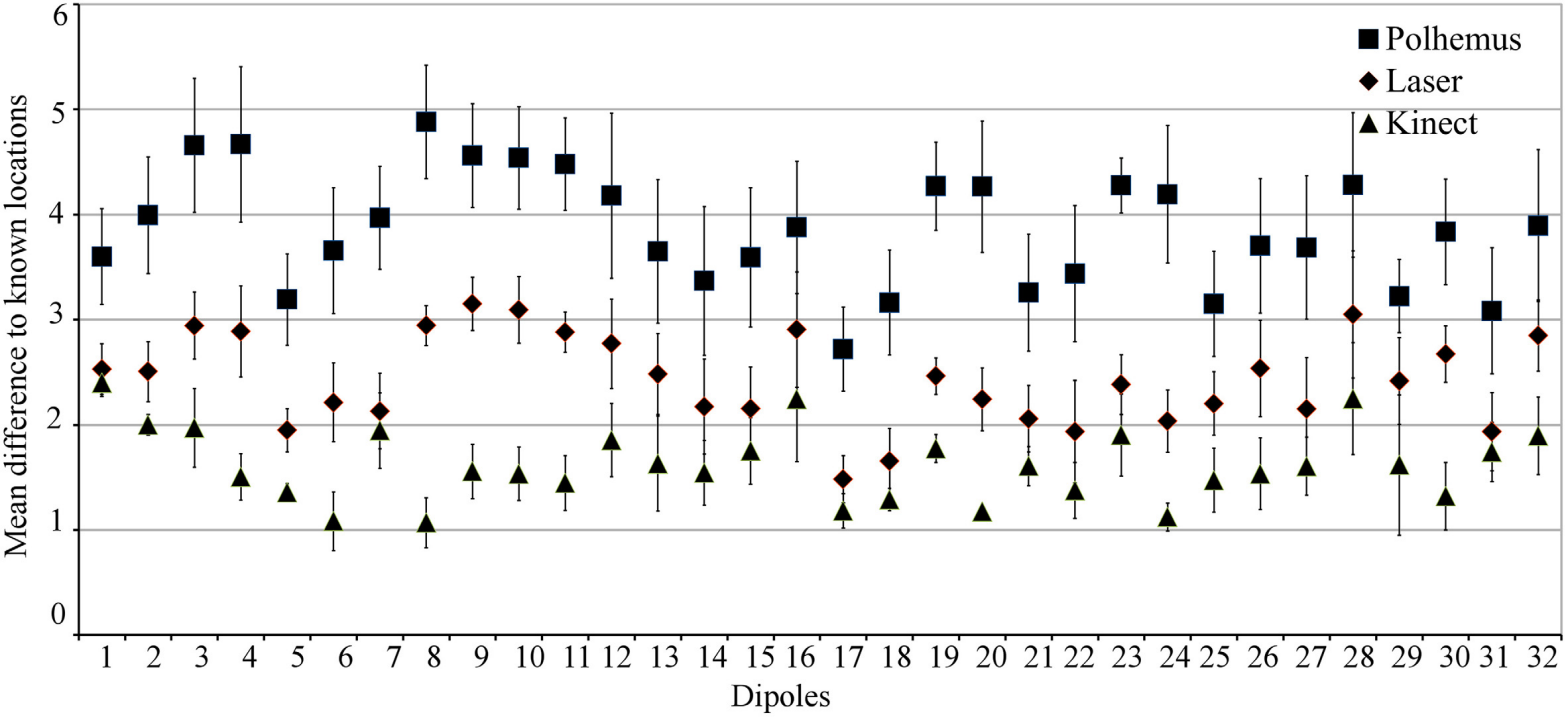
**Phantom Source Localization Accuracy**. The mean difference from the known current source location is shown for each digitization device. Error bars indicate standard error.

Figure 2 shows the mean displacement between the known and estimated location of each current source in the calibrated phantom across all three digitization systems. This provides the profile of source localization accuracy for each device across a number of locations in the calibrated phantom. The Kinect sensor outperforms the Polhemus and laser scanner at all 32 locations for accurately estimating the current source location. Across all calibrated sources, the mean source localization accuracy and standard error for the Polhemus, laser scanner, and Kinect are 3.85 ± 0.57, 2.43 ± 0.34, and 1.62 ± 0.28, respectively. Thus, the Kinect improved the source localization accuracy over the Polhemus and laser scanner by 2.23 mm and 0.81 mm, respectively. Investigating these results in the cardinal planes showed that the source localization error for laser and Polhemus was dominant in the z-direction (i.e., inferior–superior).

ANOVA results showed that there was a main effect of digitization device, indicating that source localization accuracy was significantly different depending on which device was used. *Post-hoc* testing indicated that this main effect was due to the Kinect scanner having better source localization accuracy than both the Polhemus and the laser scanner. There was also a significant interaction between device and dimension. *Post-hoc* testing indicated that this interaction was caused mainly by differences in localization accuracy in the z-direction, with the Polhemus performance significantly worse than the laser, and the laser performance significantly worse than the Kinect.

### Human study

**Figure 3:**
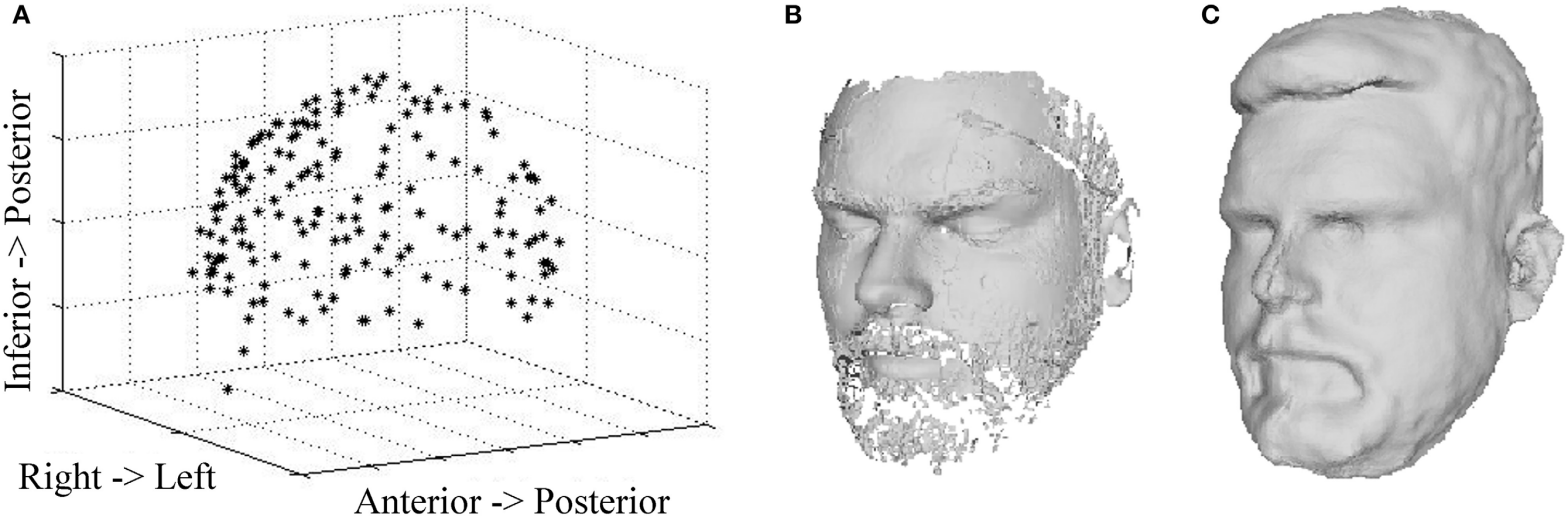
**Head Surface Digitization Data**. 3-D reconstruction data are shown for a representative participant based on **(A)** Polhemus scan, **(B)** laser scan, and **(C)** Kinect scan.

Figure 3 shows the digitization data from all three digitization devices for a representative subject. The Polhemus digitization system captured 150 points around the participant's head. When combined into a single scan, the three, 60-s laser scans included 100,000 ± 10,000 points, including HPI coils and the anatomical landmarks generally used for MEG-MRI co-registration (e.g., nasion, pre-auricular points). The continuous Kinect scan captures a 3-D image with 310,000 ± 30,000 points in less than a minute. During acquisition, the complete image is visible in the Fusion software, allowing the operator the opportunity to acquire additional points as necessary. As compared to the Polhemus digitization, other facial structures (cheeks, eyes, forehead, and nose) are clearly visible in the laser and Kinect images, providing further constraints for co-registration with MRI.

**Figure 4:**
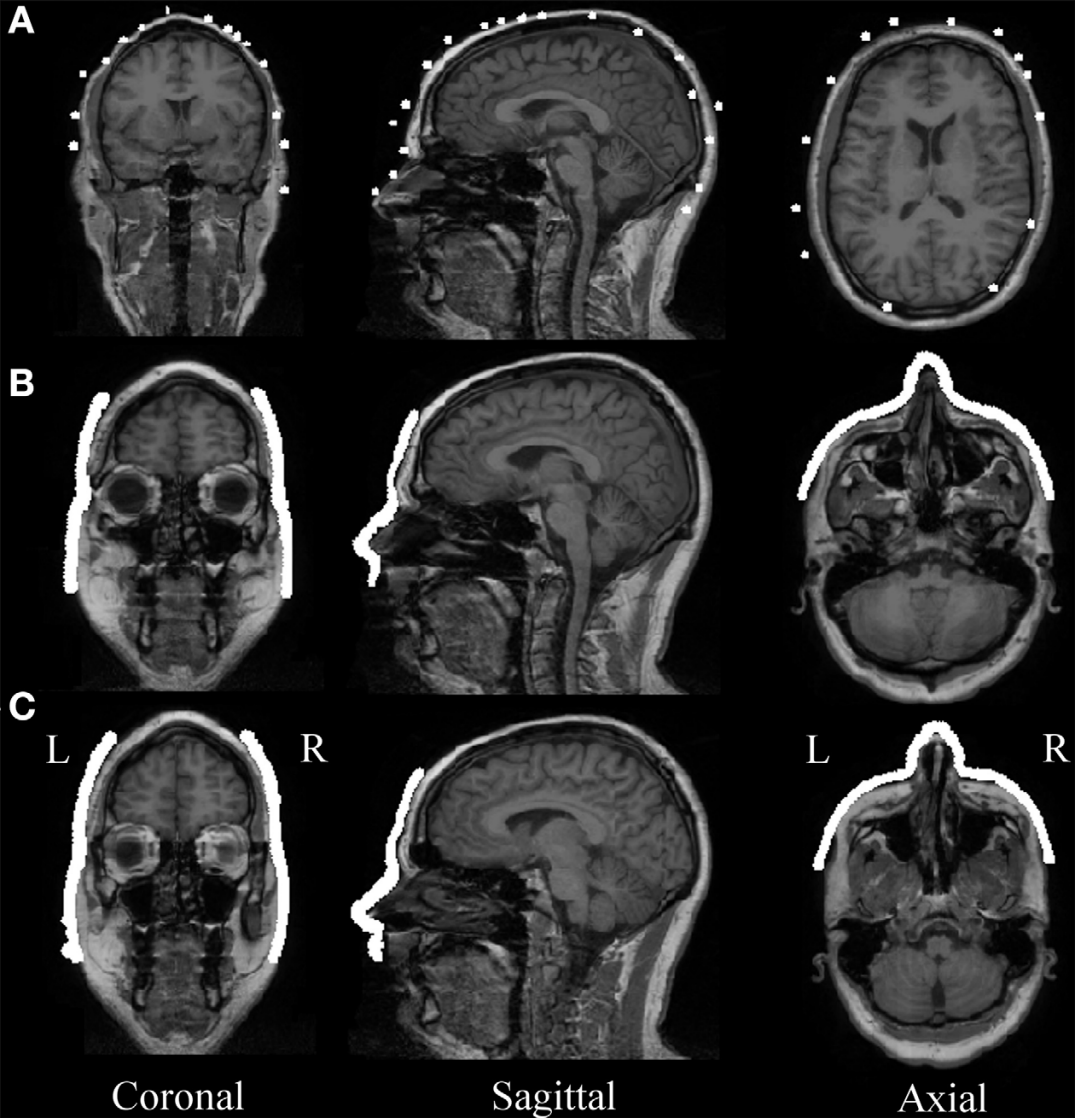
**MEG-MRI Co-registration. (A)** Polhemus, **(B)** laser scanner, and **(C)** Kinect scanner head digitization data are overlaid on the MR image for a representative participant, after automatic co-registration. Different slice is used for Polhemus to highlight co-registration accuracy in slices that included the most digitization points.

Automatic MEG/MRI co-registration with the ICP algorithm was successful for all of the five participants and all three digitization devices. The laser and Kinect face data after it was restricted to above the upper lip and below the hairline had about 21,000 ± 2000 and 17,000 ± 1000 respectively (mean ± standard error). The quality of automated MEG-MRI co-registration for all three devices is visualized as a superposition of the aligned digitization data on the MRI in Figure 4. The same representative participant is displayed as in Figure 3. While all three devices provide a reasonable co-registration, the increased point density with the laser and Kinect digitization devices improves the capacity for aligning anatomical landmarks. The mean co-registration accuracy is summarized in Table 1. Across the group, MEG-MRI co-registration accuracy following automated alignment was equivalent between the laser and Kinect digitization systems (2.2 mm), but poorer for the Polhemus (5.1 mm). ANOVA results showed that there was a main effect of digitization device, indicating that there were significant differences in the MEG-MRI co-registration error between the three devices. *Post-hoc* testing revealed that the MEG-MRI co-registration was not significantly different between the Kinect and laser scanner. However, the MEG-MRI co-registration accuracy was significantly better for the Kinect, as compared to the Polhemus.

**Table 1:** **MEG-MRI co-registration accuracy with ICP**.

**Subject**	**Co-registration accuracy (mean ± standard error; mm)**		
	**Polhemus**	**Laser**	**Kinect**
1	5.55±0.51	2.62±0.017	2±0.012
2	5.06±0.66	1.98±0.013	2.27±0.018
3	5.51±0.73	2.06±0.011	2.31±0.016
4	2.92±0.31	2.06±0.012	2.15±0.013
5	6.3±0.92	2.3±0.016	2.38±0.017
**Mean ± Standard Error**	5.07±0.57	2.2±0.11	2.22±0.066

## Discussion

The accurate localization of brain areas that generate activity in MEG is dependent on the MEG-MRI co-registration accuracy, which is in turn dependent on the digitization of the head shape. In our previous work (Bardouille et al., 2012), we showed a laser scanner provided better surface and source localization accuracy in comparison to the commonly used Polhemus device. Further, we showed that the laser scanner provided better MEG-MRI co-registration accuracy. However, consumer-grade laser scanner systems currently require the participant to remain still for 60 seconds during scanning, which limits applicability. In the present work, we compared the performance of the Kinect scanner, which does not suffer from this limitation, to the Polhemus and laser scanner. Scans with a calibrated phantom showed that the Kinect digitization process improved the phantom source localization accuracy by 137 and 50% when compared to Polhemus and laser devices, respectively. Significantly, this result reflected a 2.23 mm and 0.81 mm average improvement in source localization accuracy when compared to Polhemus and laser, respectively. Human scans with all three devices showed that the Kinect provided equivalent MEG-MRI co-registration accuracy, as compared to the laser scanner, and that both devices out-performed the Polhemus. These results suggest that the Kinect scanner can be used to improve localization accuracy in MEG scanning without reducing access to difficult participants.

One important advantage of the Kinect scanner is that the device collects 2000 times more points than the Polhemus and three times more points than the laser scanner in one third of the time. Reducing scan time is important in neuroimaging to ensure that medical devices are used as efficiently as possible. In many cases, reduction in scan time occurs with some trade-off in scan quality. In this case, the Kinect scanner provides more data with improved scan quality in less time. Further, due to the density of surface points, the facial features are captured with high resolution, which helps in automatically co-registering with the anatomical MRI using the ICP algorithm. Automatic co-registration using laser and Kinect scans leads to an additional reduction in data analysis time, and opens the door for immediate quality assurance, given access to a high resolution MRI with good coverage of facial features prior to the MEG scan.

An additional advantage of the Kinect scanner is the vendor-supplied Fusion software, which enables the real-time integration of individual digitization frames acquired at up to 30 frames per second. Specifically, the Kinect Fusion software tracks the movement of the participant's head during the digitization process. This functionality, which exists with the Polhemus but is not possible using the laser scanner, potentially increases the utility of the device for scans with participants who have difficulty staying still (e.g., some patient populations, children). Further, the Fusion software displays the captured 3-D head shape in real-time, providing immediate feedback to the operator to scan any missing regions in the captured model. Again, this was not feasible with the laser scanner, as 3-D head shapes can only be viewed after the each 60 s scan was completed. In the case of problematic laser scans, the entire 1 min scan must be repeated.

The Fusion software also provided the opportunity for the development and implementation of adaptations to the real-time digitization process. The implementation of the erosion algorithm improved the co-registration of color and depth data near the edges of objects in the Kinect's field of view. This led to better representation of the stickers used for identification of HPI coils and anatomical landmarks.

## Conclusion

Three-dimensional head shape digitization for MEG scanning using the Kinect device improves the source localization accuracy for a calibrated phantom by 2.23 mm and 0.81 mm with respect to the Polhemus and laser scanners, respectively. The digitization sampling rate (points per unit time) is also greater with the Kinect, when compared to the Polhemus and laser scanners. The head digitization data is acquired by the Kinect in less time than either the Polhemus or laser scanner, and will co-register scans in real-time at a rate of 30 frames per second. Real-time scan alignment has the potential to help in scanning populations that have difficulty remaining still. The mean accuracy in co-registering MEG and MRI data in human participants using the Kinect was improved by 2.85 mm with respect to the Polhemus device and was similar to the same measure for the laser scanner. As such, the Kinect scanner is a viable option for improving localization accuracy in MEG scanning while maintaining access to difficult patient populations.

### Conflict of interest statement

Funding for this project was provided by an award from the Atlantic Canada Opportunities Agency's Atlantic Innovation Fund, with matching funds provided by Elekta Neuromag Oy.
